# The Dilemma of Lithium Discontinuation in Bipolar Disorder During Pregnancy Planning: A Case Report and Literature Review

**DOI:** 10.1192/j.eurpsy.2024.888

**Published:** 2024-08-27

**Authors:** B. Orgaz Álvarez, E. Jiménez Sola, M. Velasco Santos, P. Ibáñez Mendoza, Á. de Vicente Blanco, S. Cebolla Lorenzo

**Affiliations:** Hospital Universitario La Paz, Madrid, Spain

## Abstract

**Introduction:**

Lithium is considered the gold standard mood stabiliser for bipolar disorder, yet its use during pregnancy remains controversial, demanding careful consideration of potential risks and benefits. Classically, it has been associated with an increased risk in congenital heart defects, however, recent studies point towards a much lower absolute risk than was previously believed. Furthermore, discontinuation of lithium before or during pregnancy poses a high risk of destabilisation and lithium has been shown to reduce the risk of relapse both in pregnancy and in the postpartum period. Hence, treatment planning is of the upmost importance in this patient group and individual risk stratification should be undertaken for patients to make informed decisions about their treatment.

**Objectives:**

To describe the case of a patient with bipolar disorder who discontinued lithium treatment while attempting to conceive and subsequently presented with a manic episode and to expand the scientific knowledge on this topic.

**Methods:**

Case report and brief literature review.

**Results:**

A 41-year-old patient with a diagnosis of bipolar disorder, previously on lithium 900mg/day, was admitted to the emergency department with symptoms suggestive of a manic episode. One month prior, the patient had discontinued treatment with lithium due to her desire to pursue pregnancy and interrupt treatment while trying to conceive. The patient had a history of postpartum psychosis followed by various depressive and manic episodes with psychotic symptoms, leading to a bipolar disorder diagnosis and commencing treatment with lithium. Her consultant psychiatrist had informed her of the individualised risks of interrupting treatment with lithium and had advised to continue treatment alongside frequent follow-up due to the high-risk of relapse. Despite her consultant’s recommendation, she decided to interrupt treatment and hence a personalised lithium tapering regime and advice to continue treatment with quetiapine 200mg/day was given.

During the ED stay, treatment with olanzapine was introduced which helped to stabilise her symptoms. Lithium levels were subtherapeutic (lithium serum level 0.11 mmol/L). Inpatient psychiatric admission was avoided due to rapid symptom improvement, strong social support in the community and her preference for ambulatory care. Lithium was gradually reintroduced and antipsychotic treatment was adjusted at follow up appointments, which ultimately led to the resolution of symptoms and stabilisation.

**Conclusions:**

This case highlights the significance of considering continuing lithium treatment in bipolar disorder during pregnancy planning. Decisions about medication in pregnancy are multifaceted, making appropriate risk stratification imperative in order to inform individualised care plans to minimise the risk of relapse in these patients.

**Disclosure of Interest:**

None Declared

